# Comparison of General Surgical Practice Size and Setting in 2017 vs 2013 in the US

**DOI:** 10.1001/jamanetworkopen.2021.6848

**Published:** 2021-04-28

**Authors:** Thomas C. Tsai, Benjamin H. Jacobson, Evan M. Benjamin, Jose F. Figueroa

**Affiliations:** 1 Department of Health Policy and Management, Harvard Chan School of Public Health, Boston, Massachusetts; 2 Department of Surgery, Brigham and Women’s Hospital, Boston, Massachusetts; 3 Ariadne Labs, Boston, Massachusetts; 4 Division of Medicine, Brigham and Women’s Hospital, Boston, Massachusetts

## Abstract

**Importance:**

As health care delivery markets have changed and new payment models have emerged, physicians in many specialties have consolidated their practices, but whether this consolidation has occurred in surgical practices is unknown.

**Objective:**

To examine changes in the size of surgical practices, market-level factors associated with this consolidation, and how place of service for surgical care delivery varies by practice size.

**Design, Setting, and Participants:**

A cross-sectional study of Medicare Data on Provider Practice and Specialty from January 1 to December 31, 2013, compared with January 1 to December 31, 2017, was conducted on all general surgeon practices caring for patients enrolled in Medicare in the US. Data analysis was performed from November 4, 2019, to January 9, 2020.

**Exposures:**

Practice sizes in 2013 and 2017 were compared relative to hospital market concentration measured by the Herfindahl-Hirschman Index in the hospital referral region.

**Main Outcomes and Measures:**

The primary outcome was the change in size of surgical practices over the study period. Secondary outcomes included change in surgical practice market concentration and the place of service for provision of surgical care stratified by surgical practice size.

**Results:**

From 2013 to 2017, the number of surgical practices in the US decreased from 10 432 to 8451. The proportion of surgeons decreased in practices with 1 (from 26.2% to 17.4%), 2 (from 8.3% to 6.6%), and 3 to 5 (from 18.0% to 16.5%) surgeons, and the proportion of surgeons in practices with 6 or more surgeons increased (from 47.6% to 59.5%). Hospital concentration was associated with an increase in the size of the surgical practice. Each 10% increase in the hospital market concentration was associated with an increase of 0.204 surgeons (95% CI, 0.020-0.388 surgeons; *P* = .03) per practice from 2013 to 2017. Similarly, a 10% increase in the hospital-level HHI was associated with an increase in the surgical practice HHI of 0.023 (95% CI, 0.013-0.033; *P* < .001). Large surgical practices increased their share of Medicare services provided from 36.5% in 2013 to 45.6% in 2017. Large practices (31.3% inpatient in 2013 to 33.1% in 2017) were much more likely than small practices (19.0% inpatient in 2013 to 17.7% in 2017) to be based in hospital settings and this gap widened over time.

**Conclusions and Relevance:**

Surgeons have increasingly joined larger practices over time, and there has been a significant decrease in solo, small, and midsize surgical practices. The consolidation of surgeons into larger practices appears to be associated with hospital market concentration in the same market. Although overall care appears to be more hospital based for larger practices, the association between the consolidation of surgical practices and patient access and outcomes should be studied.

## Key Points

**Question:**

What was the change in consolidation and place of service for US surgical practices from 2013 to 2017?

**Findings:**

Using a cross-sectional analysis of Medicare practice and claims data, changes toward concentration of surgeons in practices of a larger size were noted, from 24 958 general surgeons in 10 432 practices in 2013 to 26 250 general surgeons in 8451 practices in 2017. Highly concentrated hospital markets were associated with an increase in practice size, and large practices were increasingly based in inpatient settings.

**Meaning:**

These findings suggest that surgeons are consolidating into larger practices; the association with patient access and outcomes should be studied.

## Teaser

This cross-sectional study examines changes in the number of general surgeons and consolidation of surgical practices from 2013 to 2017.

## Introduction

The landscape of the US health care delivery system is rapidly changing. Substantial attention has been paid to the consolidation of hospitals into health systems, and there has been a similar alteration in vertical integration through the purchasing and merging of physician practices.^1,2^ Research suggests that physicians across a number of specialties are joining health systems or consolidating into larger physician practices.^3,4^ This consolidation is especially true for procedural specialists, with 40% of general surgical practices owned by a hospital or a health system.^5^ In light of the financial pressures on surgical practices during the COVID-19 pandemic, there is concern that these trends may be particularly pronounced for surgeons.

These changes may have been further accelerated by the transition to value-based care as part of recent health reform efforts. Many of these new quality-improvement and value-based efforts have increased administrative burden, reduced physician leverage, and heightened the challenges of successfully operating a small or solo practice.^6^ There is concern that alternative payment models, such as accountable care organizations and bundled payments, which reward coordination of care and use management, may have accelerated the consolidation of surgical practices.

The change of the size of US surgical practices in response to these new pressures has not been well studied, yet it is of importance in understanding the ability of the US surgical delivery system to maintain access to high-quality surgical care for patients. Furthermore, little is known about the market-level factors that may be associated with the consolidation of surgical practices. It is possible that supply-side factors, such as the availability of primary care physicians, or community sociodemographic factors may influence the market and therefore inform decisions among surgeons to join larger practices. General surgeons, who often take trauma or emergency general surgery call at hospitals, may be especially sensitive to market forces of hospital market concentration and vertical integration. Despite the evidence that surgical practices are more increasingly affiliated with health systems, to our knowledge, there has not been a systematic study of whether the place of service of the surgical care that is provided has correspondingly changed.

Therefore, to provide empirical data on these changes, we sought to answer the following questions. First, are surgeons increasingly practicing in larger practices? Second, are market-level factors associated with surgeons consolidating into larger practices over time? Third, how does the place of service—the site of clinical care delivery—differ between large and small surgical practices? We hypothesized that surgical practices have increased in size in response to consolidation of the hospitals in a given market. In addition, we hypothesized that a shift has occurred in surgical care from the office-based to the inpatient setting.

## Methods

### Data Sources

We examined surgical practices using the Medicare Data on Provider Practice and Specialty (MD-PPAS) from January 1 to December 31, 2013, and January 1 to December 31, 2017. Data analysis was conducted from November 4, 2019, to January 9, 2020. The MD-PPAS data set contains physician-level information including age, sex, and specialty.^7^ These data were obtained from the Medicare Provider Enrollment, Chain, and Ownership System and are validated by the Centers for Medicare & Medicaid through linkage of those data with Medicare claims. The data set distinguishes between physicians in group or individual practices by determining whether a physician files any claims with a tax identification number (TIN) for a group practice. This study was limited to general surgeons, which was defined by National Provider Identifiers (NPIs) associated with general surgery, colorectal surgery, and surgical oncology specialties. Information on health systems was obtained from the Agency for Healthcare Research and Quality Compendium of US Health Systems from 2018.^2^ This project was approved and a waiver of informed consent was granted by the institutional review board of the Harvard T.H. Chan School of Public Health. This study followed the Strengthening the Reporting of Observational Studies in Epidemiology (STROBE) reporting guideline.

Hospital market variables were obtained from the Dartmouth Atlas of Healthcare, which reports data at the hospital referral region (HRR) level.^8^ Hospital referral regions are geographic areas that represent health care markets. Market concentration was calculated using the Herfindahl-Hirschman Index (HHI). Our primary indicator was hospital-level HHI, which was calculated by summing the squares of each hospital’s market share within each HRR, as determined by discharge totals from the Centers for Medicare & Medicaid Hospital Service Files. The HHI score ranges from 0 to 1, with higher numbers indicating more concentrated markets and lower numbers indicating more competitive ones. Our primary outcome variable was the change in the average number of surgeons per practice at the HRR level. Our secondary outcome variable was the surgical practice–specific HHI, calculated by summing the squares of each surgical practice share within an HRR for corresponding years. Community-level data were taken from American Fact Finder using the 5-year (2011 to 2015) American Community Survey and was aggregated from zip codes to HRRs using population-weighted averages.^9^

### Statistical Analysis

We identified US practices with at least one surgeon in 2013 and 2017. To account for surgeons billing under multiple group practices, we weighted each surgeon by 1 divided by the number of group practices they billed over, similar to the method used by Muhlestein and Smith^4^ to avoid overcounting surgeons who may bill under multiple practices. We then determined the number of practices with 1 (solo), 2 (small), 3 to 5 (medium), and 6 or more surgeons (large). We further determined the proportion of all surgeons practicing in the US who were in each practice size category. We next determined the proportion of Medicare claims from the 20% sample attributed to surgical practices by aggregating claims associated with surgeon NPI into surgical practices by assignment based on practice TIN.

We used a multivariable linear regression model to investigate the association between hospital market concentration and our primary outcome variable, the change in mean surgeons per practice from 2013 to 2017. Market-level factors included supply-side factors (hospital-level HHI, baseline surgical practice–level HHI, physicians per 1000 people, primary care physicians per 1000 people, hospital beds per 1000 people, nurses per 1000 people, and Medicare spending per capita), community-level factors (total population of market, total Medicare population of market, proportion of market of individuals of White race, median income of the market, and proportion of the population in market below the federal poverty line), and physician-level factors (mean age and proportion of women). Because growth in the average size of the practice may not necessarily imply increased surgical practice–market concentration if each practice grew at a similar rate, we then assessed the association between hospital and surgical market concentration using a second multivariable linear regression model with change in the surgical practice HHI as the dependent variable.

We then categorized claims by the place of service codes to assess for changes in place of service for surgical care delivery as a function of practice size between 2013 and 2017. The site of clinical care delivery was categorized by claim-level place of service codes in line with current Centers for Medicare & Medicaid terms to include office, hospital inpatient, hospital outpatient, emergency department, and ambulatory surgical center locations.

Findings were considered statistically significant at *P* = .05 with unpaired, 2-sided testing. Statistical analyses were performed using SAS, version 9.4 (SAS Institute Inc).

Although detailed data on the hierarchical relationship between surgical practices and health systems were not fully available for all years of our study, we merged the 2017 surgical practice–level data with the 2018 Agency for Healthcare Research and Quality Compendium of US Health Systems to assess the affiliation of practices into health systems. We further assessed for the geographic variation in increase in surgeon practice market concentration across HRRs.

## Results

**Figure 1.**
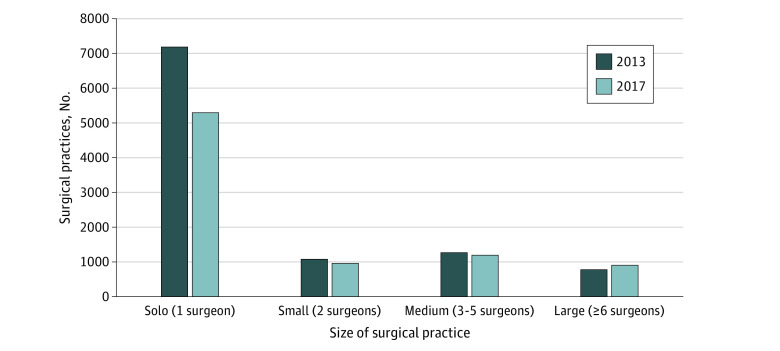
Changes in Practice Size From 2013 to 2017

**Figure 2.**
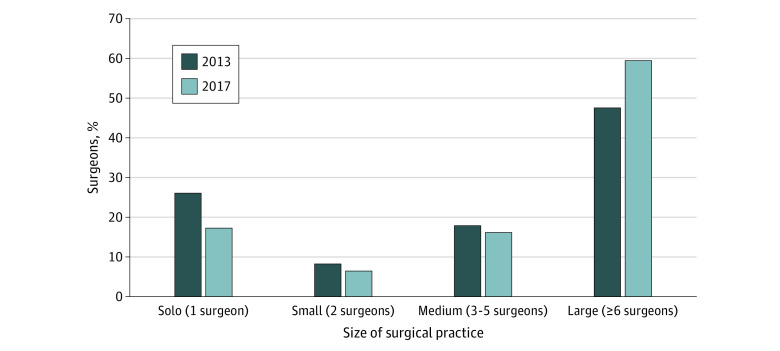
Changes in Proportion of Surgeons From 2013 to 2017

**Figure 3.**
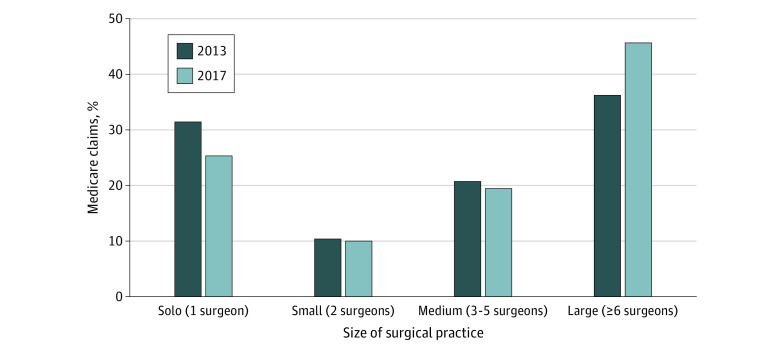
Changes in Proportion of Medicare Claims From 2013 to 2017

In 2013, there were 24 958 general surgeons in 10 432 practices providing surgical care for Medicare beneficiaries. In 2017, the number of surgeons increased to 26 250 surgeons and the number of practices decreased to 8451 practices. Approximately 85% of surgeons for both years were affiliated with only a single practice (eTable 1 in the Supplement). The number of solo surgical practices decreased by 26% (from 7188 to 5308). Decreases were also noted in small practices with 2 surgeons (from 1124 to 968) and medium practices with 3 to 5 surgeons (from 1301 to 1232). Conversely, large practices with 6 or more surgeons increased over this 4-year period (from 819 to 943) (Figure 1). Correspondingly, the proportion of surgeons in solo practices (from 26.2% to 17.4%), small practices (from 8.3% to 6.6%), and medium practices (from 18.0% to 16.5%) decreased, and the proportion of surgeons in large practices increased (from 47.6% to 59.5%) (Figure 2). This shift of surgeons to larger practices was matched by a similar shift in claim volume. In 2013, 31.8% of Medicare surgical claims came from solo surgical practices and 36.5% came from large practices. In 2017, however, only 25.4% of claims came from solo practices and 45.6% were from large practices (Figure 3).

Hospital market concentration was associated with an increase in surgical practice size, although no other supply-side, community-level, or physician-level factors were associated with a change in surgical practice size from 2013 to 2017 (Table; eTable 2 in the Supplement). A 10% increase in the hospital-level HHI was associated with an increase of 0.204 (95% CI, 0.020-0.388; *P* = .03) surgeons per practice from 2013 to 2017. Similarly, a 10% increase in the hospital-level HHI was associated with an increase in the surgical practice HHI of 0.023 (95% CI, 0.013-0.033; *P* < .001).

**Table.** Association of Selected Market-Level, Community-Level, and Physician-Level Factors With Change in Mean Surgeon Practice Size and Surgical Practice HHI, 2013 vs 2017

Market-level and community-level factor	Change in mean surgeons per surgical practice (95% CI)	*P* value	Change in surgical practice HHI (95% CI)	*P* value
Hospital market concentration per 10% increase in HHI	0.204 (0.020-0.388)	.03	0.023 (0.013-0.033)	<.001
Proportion of White population	−0.600 (−2.812 to 1.613)	.59	0.001 (−0.120 to 0.122)	.99
Primary care physicians per 1000 people	0.009 (−0.031 to 0.048)	.67	0.000 (−0.002 to 0.002)	.78
Proportion of population below federal poverty line	0.040 (−0.058 to 0.138)	.43	0.002 (−0.003 to 0.008)	.38

Abbreviation: HHI, Herfindahl-Hirschman Index.

**Figure 4.**
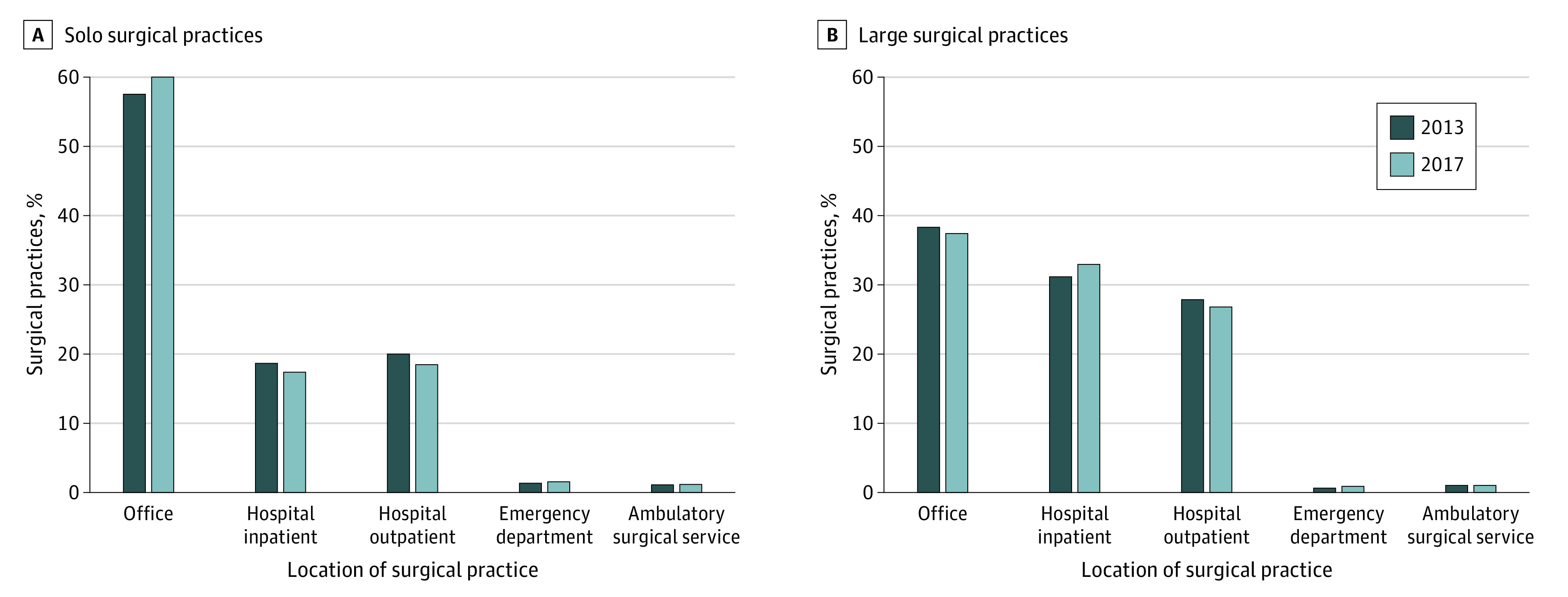
Changes in Practice Base From 2013 to 2017

In both 2013 and 2017, a greater proportion of solo surgical practices were based in an office setting than large surgical practices; considerably more large surgical practices were based in hospital inpatient settings than solo surgical practices (Figure 4). This gap widened over the 4-year period, with solo surgical practices further shifting from hospital inpatient (from 19.0% to 17.7%) to office (from 57.6% to 60.2%) settings; however, practices with greater than 5 surgeons moved from office (from 38.4% to 37.5%) to hospital inpatient (from 31.3% to 33.1%) settings.

### Sensitivity Analyses

When merged with data from the 2018 Agency for Healthcare Research and Quality Compendium on US Health Systems, 21% of the general surgical practices were identified as integrated into 583 health systems. There was a mean (SD) of 3.0 (5.6) general surgical practices associated with each health system. General surgical practices associated with health systems were larger, with a mean (SD) of 8.3 (14.9) surgeons per practice compared with 1.7 (3.0) surgeons in practices not integrated with health systems (difference of 6.5 surgeons per practice: 95% CI, 6.2-6.9; *P* < .001). There was significant geographic variation in change in surgeon-practice market concentration (eFigure in the Supplement).

## Discussion

From 2013 to 2017, there was a clear shift toward surgical practice consolidation, with 3 of 5 surgeons practicing in large practices. Solo, small, and midsize practices all decreased during that time, and larger practices with 6 or more surgeons increased. In contrast to the 22% decrease in the number of solo practices, there was a 15% increase in the number of large practices. Controlling for community- and physician-level factors, hospital market concentration was associated with the increase in surgical practice size. In addition, we found that large practices were more likely to be based in hospital settings than smaller practices, and the proportion of large practices in hospital settings has grown while smaller practices have shifted further toward an office-based setting.

Overall, we found a growth in the number of general surgeons, but this growth was primarily concentrated in large practices of 6 or more surgeons. Our estimates of the number of surgeons using the new MD-PPAS data set is consistent with general surgery workforce estimates from the existing literature using data from the Association of Academic Medical Centers, the American Medical Association Masterfile, or the US Census Current Population Survey.^10,11,12^ Our results show that changes in the consolidation of general surgery practices match similar changes in physician practice consolidation across other medical specialties.^3^ Surgeons running small practices may find it difficult to compete with larger practices affiliated with health systems. Furthermore, when a hospital is purchased by a larger health system, surgeons in small practices based within that hospital may be forced to join a larger physician organization within the health system or relocate their practice to remain independent.^13,14^ More so than in many specialties, surgery has specific infrastructure, technological, and equipment requirements, and the choice to relocate a practice may be especially burdensome. Therefore, health care systems hold considerable leverage in consolidating surgical practices as they expand their hospital networks.

We further found a significant association between hospital market concentration and increase in surgical market concentration, as more highly concentrated hospital markets were associated with an increase in both the number of surgeons per practice and in the surgical practice–level HHI. The Department of Justice considers a transaction that increases the market HHI by 0.01 in moderate or highly concentrated markets as possible for raising anticompetitive concerns and therefore warranting evaluation by the Federal Trade Commission.^15^ Therefore, our finding of a 10% increase in hospital market HHI associated with a 0.02 increase in surgical practice HHI is substantial from the standpoint of federal regulation on the horizontal integration of physician practices. Because we could not assess ownership of surgical practices by health systems, whether the observed increase in surgical practice market concentration was due to vertical integration of multiple practices into health systems or horizontal integration of multiple practices is unknown but warrants further study.

One hypothesis for the alterations in surgical practice consolidation has been the response to alternative payment models. Accountable care organizations and bundled payment models have ushered in a new era of coordinated care and use management associated with an ever-increasing burden of reporting use and quality metrics. This proliferation of quality measurement has resulted in a high cost to the health care system.^16^ Practices must confront new requirements and regulations that, although improving the quality and efficiency of care for patients, require substantial time and financial resources from the physicians. Surgeons in solo or small practices may find it challenging to continue in this current landscape and might choose to join larger practices to distribute the financial and administrative costs of operating a surgical practice today.^17^ These issues may also have contributed to the change in reported physician burnout.^18^ Despite these concerns, emerging data suggest that the movement toward practice consolidation is independent of enrollment in accountable care organizations and seems largely responsive to secular market variations.^19^ Even with the shift toward larger surgical practices, we still found that 1 in 5 surgeons worked in a solo practice as of 2017. Given the large financial impact of COVID-19 associated with the financial viability of small practices, it remains unknown whether the decline in solo or small surgical practices will be accelerated as a result of the COVID-19 pandemic or balanced by an increased desire for practice autonomy.^20^

The decision to join a larger surgical practice may also be associated with a desire to increase market power. Concentration of larger health insurers has decreased physician leverage and reduced earnings compared with physicians in areas of less substantial insurer competition.^21^ As a result, physicians in small or solo practices may find it challenging to compete with these larger, more concentrated organizations. This market reality may well push surgeons to consolidate into larger practices to increase bargaining power for reimbursements. There is emerging evidence that, similar to hospital consolidation, physician consolidation may result in higher prices and greater out-of-pocket spending for patients.^22^ We observed a substantial increase in the proportion of Medicare claims arising from large surgical practices over the same period as the shift of surgeons into these larger practices, suggesting an increase in market power. The effect that practice consolidation may have on clinical outcomes for patients remains unknown. The literature on hospital consolidation indicates possible harm in terms of both increased complications for some surgical procedures and decreased patient satisfaction, and whether these patterns also apply to consolidation of physician practices warrants further study.^23,24^ Our findings, therefore, provide baseline data for future studies on the associations between surgical practice consolidation and outcomes for surgical patients.

### Limitations

This study has several limitations. First, the data available in MD-PPAS only include physicians who are enrolled in Medicare. As such, these results may not extend to surgeons who do not see patients with Medicare coverage. Second, physicians are grouped into practices by TIN and larger practices may report using multiple TINs, which could affect actual group size. Given that all practices with 6 or more surgeons are grouped, however, this risk is likely mitigated to a large extent. Third, we defined the market by the hospital referral region. Although this is a common market definition in health services research, other market definitions, such as hospital service areas or the Pittsburgh Atlas emergency and trauma care referral regions, could also be used for an analysis of surgical practices.^25,26^ However, because HRRs were originally based on tertiary care, including referral patterns for surgical procedures, HRRs provide a meaningful definition of the market from the perspective of surgical practices. Because hospitals have closed or consolidated into larger health systems, HRRs may not be fully representative of hospital referral patterns in the contemporary era.^2^ We also could not fully account for hierarchical associations across surgeons, practices, and health systems within an HRR. Future analyses of physician practice consolidation should consider the complex hierarchical structure of both vertically and horizontally integrated health systems. In addition, this study only examined surgeons who practice with other surgeons. Although this specification suited our goal of examining whether surgeons are increasingly joining with other surgeons, it does not consider the effects of multispecialty practices. Previous studies have reported that oncologists, cardiologists, and other specialists are also consolidating, so multispecialty consolidation may be an important factor to consider in future work.^3,4,5,27^

## Conclusions

Surgical practices are consolidating over time and shifting from solo and small practices to practices with greater numbers of surgeons. The association between hospital consolidation and surgical practice consolidation, as well as the shift of larger surgical practices toward inpatient hospital settings, suggests that vertical integration of hospitals may play a major role in motivating surgeons to join larger practices. Further research is needed to assess whether this consolidation has had a positive or detrimental association with access and outcomes for patients.

## Notes

Supplement. **eTable 1.** Distribution of Surgeons by Number of Practice Affiliations **eTable 2.** Association of Market, Community, and Physician Level Factors With Change in Mean Surgeon Practice Size and Surgical Practice HHI **eFigure.** Geographic Variation in Change in Surgical Practice HHI from 2013 to 2017
