# Supplementary material for: Comparison of General Surgical Practice Size and Setting in 2017 vs 2013 in the US
Source: JAMA Netw Open. 2021 Apr 28;4(4):e216848. doi: 10.1001/jamanetworkopen.2021.6848 (PMC8082320; doi:10.1001/jamanetworkopen.2021.6848)
Supplement: Supplement. — eTable 1. Distribution of Surgeons by Number of Practice Affiliations eTable 2. Association of Market, Community, and Physician Level Factors With Change in Mean Surgeon Practice Size and Surgical Practice HHI eFigure. Geographic Variation in Change in Surgical Practice HHI from 2013 to 2017 [file jamanetwopen-e216848-s001.pdf]

Supplementary Online Content

Tsai TC, Jacobson BH, Benjamin EM, Figueroa JF. Comparison of general surgical practice size and setting in 2017 vs 2013 in the US. *JAMA Netw Open*. 2021;4(4):e216848. doi:10.1001/jamanetworkopen.2021.6848

eTable 1. Distribution of Surgeons by Number of Practice Affiliations

eTable 2. Association of Market, Community, and Physician Level Factors With Change in Mean Surgeon Practice Size and Surgical Practice HHI

eFigure. Geographic Variation in Change in Surgical Practice HHI from 2013 to 2017

This supplementary material has been provided by the authors to give readers additional information about their work.

© 2021 Tsai TC et al. *JAMA Network Open.*

eTable 1. Distribution of Surgeons by Number of Practice Affiliations

Number of Practice Affiliations	2013		2017	
	Number of Surgeons	%	Number of Surgeons	%
**1**	21884	87.68%	22465	85.58%
**2**	2755	11.04%	3256	12.40%
**3**	280	1.12%	415	1.58%
**4+**	39	0.16%	114	0.43%
**Total**	24958	100%	26250	100%

© 2021 Tsai TC et al. *JAMA Network Open.*

eTable 2. Association of Market, Community, and Physician Level Factors with Change in Mean Surgeon Practice Size and Surgical Practice HHI

Market-Level Factor	Change in Mean Surgeons per Surgical Practice (95% CI)	P-Value	Change in Surgical Practice HHI (95% CI)	P-Value
**Supply Side Factor**				
Hospital Market Concentration (per 10% increase in HHI)	0.204 (0.020, 0.388)	0.03	0.023 (0.013, 0.033)	<0.001
Physicians per 1,000 people	-0.003 (-0.020, 0.014)	0.72	0.000 (-0.001, 0.001)	0.66
Primary care doctors per 1,000 people	0.009 (-0.031, 0.048)	0.67	0.000 (-0.002, 0.002)	0.78
Hospital beds per 1,000 people	-0.057 (-0.763, 0.650)	0.88	0.01 (-0.029, 0.049)	0.61
Nurses per 1,000 people	-0.211 (-0.612, 0.189)	0.3	-0.015 (-0.037, 0.007)	0.18
Medicare spending per capita (per $10,000 increase)	0.000 (0.000, 0.000)	0.13	0.000 (0.000, 0.000)	0.94
**Community-Level Factors**				
Total population of market (per 100,000 increase)	0.000 (0.000, 0.000)	0.67	0.000 (0.000, 0.000)	0.68
Total Medicare population (per 10,0000 increase)	0.000 (0.000, 0.000)	0.25	0.000 (0.000, 0.000)	0.4
Proportion of White Population	-0.600 (-2.812, 1.613)	0.59	0.001 (-0.120, 0.122)	0.99
Median income of market (per $10,000 increase)	0.000 (0.000, 0.000)	0.18	0.000 (0.000, 0.000)	0.39
Proportion of population below federal poverty line	0.040 (-0.058, 0.138)	0.43	0.002 (-0.003, 0.008)	0.38
**Physician-Level Factors**				
Mean age of surgeons	-0.101 (-0.211, 0.009)	0.07	-0.004 (-0.010, 0.002)	0.22
Proportion of female surgeons	-0.015 (-0.055, 0.025)	0.47	-0.001 (-0.003. 0.001)	0.49

Legend: Multivariate regressions of relationship of hospital market concentration with change in means surgeons per practice and surgeon practice HHI from 2013 and 2017. Models control for listed covariates as well as baseline surgical practice HHI in 2013.

© 2021 Tsai TC et al. *JAMA Network Open.*

eFigure. Geographic Variation in Change in Surgical Practice HHI from 2013 to 2017

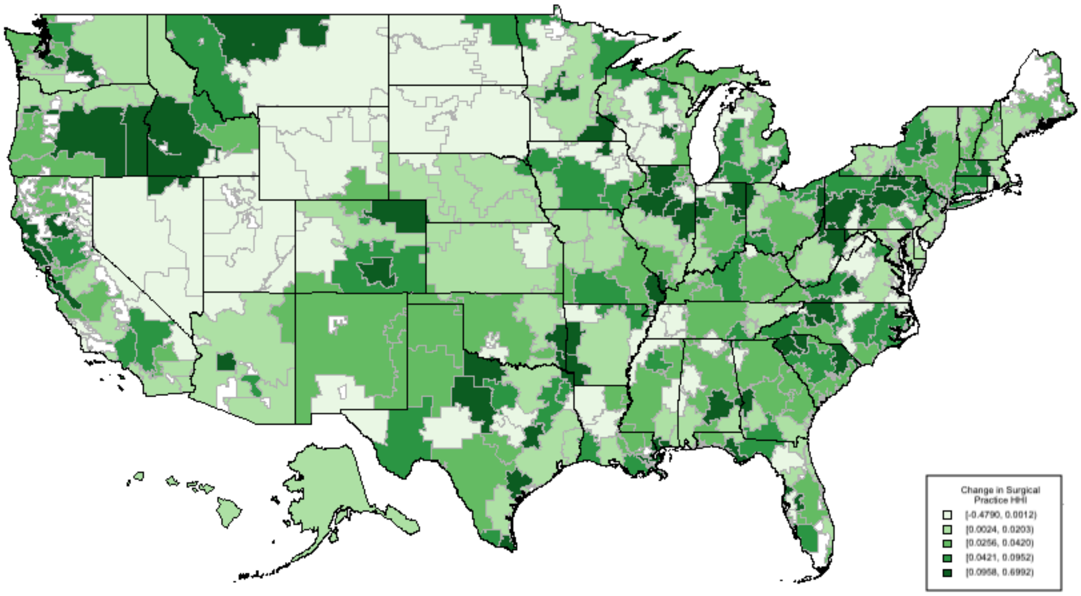

© 2021 Tsai TC et al. *JAMA Network Open.*
